# Study on Flowability Regulation of Vacuum Gas-Atomized Fe-Cr-Ni-W-B Spherical Powder

**DOI:** 10.3390/ma17061264

**Published:** 2024-03-08

**Authors:** Pengfei Yu, Jun Li, Ying Liu

**Affiliations:** School of Materials Science and Engineering, Sichuan University, Chengdu 610065, China; yupengfei@scu.edu.cn (P.Y.); lj_jun@scu.edu.cn (J.L.)

**Keywords:** Fe-Cr-Ni-W-B alloy, vacuum gas atomization, spherical powder, heat treatment

## Abstract

High-quality Fe-Cr-Ni-W-B spherical powder is crucial for the powder metallurgy preparation of high-strength and tough Fe-Cr-Ni-W-B alloys. In this study, the controlled preparation of high-quality Fe-Cr-Ni-W-B spherical powder was achieved using the vacuum gas atomization method. The effects and mechanisms of atomization gas pressure, the melt nozzle inner diameter, and heat treatment temperature on the microstructure and flowability of Fe-Cr-Ni-W-B spherical powder were systematically investigated. By optimizing process parameters, spherical Fe-Cr-Ni-W-B powder with a sphericity of 95.1% and a flowability of 15.88 s/50 g was obtained, laying the foundation for the powder metallurgy preparation of high-strength and tough Fe-Cr-Ni-W-B alloys.

## 1. Introduction

Fe-W-B system materials are a novel ternary boride with a TiNiSi crystal structure and a high content of W and B (73.4% and 4.3% wt%, respectively) [1]; these materials exhibit a wide range of unusual physical and chemical properties at elevated temperatures [2,3]. These fascinating features open a wide range of possible applications for Fe-W-B, including nuclear shielding, microwires, petrochemicals, corrosion-resistant coating, and wear-resistant materials [4,5,6,7,8,9,10,11].

Currently, casting is the most used method for preparing Fe-W-B alloys. However, the poor mechanical properties of Fe-W-B alloys seriously affect their engineering applications due to their coarse grains, the borides distributed along the grain boundaries in the form of a network, the serious segregation of alloying elements, and a large number of pores and micropores in the bulk of the material [3]. The mechanical characteristics and corrosion resistance of Fe-W-B alloys in particular are severely limited by the large contents of intermetallic borides generated due to phase transitions during the continuous cooling of the liquid phase, such as Fe_7_W_6_ and Fe*_x_*B, etc. [12,13]. The powder metallurgy technique provides several benefits over the conventional casting method, including lower production costs and a more environmentally friendly process [14,15]. Powder flowability is critical for the preparation of high-strength alloys using powder metallurgy technology [16,17]. Recent research has shown that high-performance composites can be made using powder metallurgy with spherical Fe-W-B alloy powders [1]. Therefore, the current focus lies on how to regulate the flowability of Fe-W-B alloy powders to prepare high-strength and tough Fe-W-B alloy powders.

Gas atomization is an efficient solidification technology, extensively employed by large businesses to make high-quality metal powders. Up to now, many Fe-W-B-based alloy powders have been successfully prepared by gas atomization [4]. Furthermore, recent research has shown that optimizing the shape and distribution of the ternary boride may be achieved through including chromium (Cr) elements inside the gassing Fe-W-B alloys, thus providing a solution to improve the mechanical properties [18]. Nickel (Ni) is an austenite-forming element that, when present in sufficient quantities, can increase the temperature range of the austenite phase region, allowing austenite to form at room temperature, thereby enhancing the alloy’s toughness and the steel’s hardenability. As far as we know, there are few reports regarding the preparation of Fe-Cr-Ni-W-B alloys through gas atomization. Introducing Ni elements to Fe-Cr-W-B alloys and further adopting gas atomization to prepare Fe-Cr-Ni-W-B alloy powders could potentially address the issues of ductility and corrosion resistance.

Therefore, this study aims to use gas atomization to prepare Fe-Cr-Ni-W-B powders, to systematically investigate the effects and mechanisms of atomization gas pressure, the melt nozzle inner diameter, and heat treatment temperatures on the flowability of Fe-Cr-Ni-W-B powders, and to produce alloy powders with a high flowability, thus laying the foundation for the preparation of high-strength and tough Fe-Cr-Ni-W-B alloys.

## 2. Materials and Methods

### 2.1. Preparation of Powders

Table 1 shows the raw materials and their chemical composition, which include pure iron, as well as commercially available Fe-B, Fe-W, and Fe-Cr alloys. When designing the composition, both B and W should be considered for burnout, and the design amount should fully account for this. The specific composition of the raw material was vacuum melted according to the ratio of Fe-13Cr-10Ni-xW-yB m (M = Zr, Mn, Al, etc.), where x + y = 20. Table 2 displays the specifics of these process parameters. The atomization gas pressure ranged from 3.5 to 5.0 MPa, and the inner diameter of the melt nozzle was adjusted within the range of 5.0 to 5.6 mm. The alloy powders were produced via vacuum induction melting and argon atomization processes. Place Fe-Cr, Fe-W, pure nickel, and pure iron in the melting crucible, according to the ratio of the experimental design, and put Fe-B into the charging port. Vacuum the melting bin three times, ensuring that the vacuum degree is lower than 10 Pa, and filling the bin with argon gas each time. Open the atomization bin and powder collection bin filling valves, ensuring you turn on the fan. Open the heating switch, slowly increase the temperature to 1700 °C, adjust the power to 20 kW, add Fe-B, and keep this warm for 1 min. Close the powder collecting bin inflation valve, open the high pressure inflation valve on the powder collecting bin, and close it after 5 min. After the start of the atomization process, open the valves of the powder collection bin and the filling valve, maintain a certain atomization pressure, and complete the aerosolization process of the powder.

### 2.2. Characterization

The powders were analyzed with an X-ray using a DX-2700 (Haoyuan Instrument, Dandong, China) diffractometer, equipped with a graphite monochromator and Cu K radiation, operating at 30 kV and 40 mA, with a scan rate of 0.06°/s. The powders were examined with a field emission scanning electron microscope (JSM-7900F, JEOL Ltd., Tokyo, Japan) and a transmission electron microscope (TEM, Talos F200x, Thermo Fisher Scientific, Waltham, MA, USA) to determine their shape and microstructure. An energy-dispersive X-ray spectroscopy (EDS, Oxford Instruments, Abingdon, UK) system, coupled with a scanning electron microscope and a transmission electron microscope, was used to investigate the chemical composition of powders in their micro-zones. Standard metallographic techniques were employed to generate cross-sections of the powders, and aqua regia reagent was applied in order to etch the cross-sections.

Inductively coupled plasma atomic emission spectrometry (ICP-AES, Plasma MS 300, the NCS Testing Technology, Beijing, China) was employed to analyze the chemical composition of the powder. An oxygen-nitrogen detector (TCH600, LECO, Benton Harbor, MI, USA) was utilized to determine the oxygen percentage within the powders. Particle size distribution measurements were performed on the powders using a laser particle size analyzer (Bettersize Instruments, Guangzhou, China). Image-Pro Plus (Media Cybernetics, Rockville, MD, USA) was employed to assess the sphericity and boride size of the powders. The apparent density and flowability of the powders were measured using the FL4-1 Hall flowmeter (Kunshan Guang measuring instrument equipment Co., LTD, Kunshan, China) in accordance with the Chinese national standard GB/T-1479-1984 [19]. The thermal conductivity and the diffusion coefficient of this alloy were both measured using a laser conductometer (LFA447, Netzsch Geratebau GmbH, Selb, Germany).

## 3. Results and Discussion

### 3.1. The Influence of Atomization Gas Pressure on the Flowability of the Fe-Cr-Ni-W-B Powder

**Figure 1:**
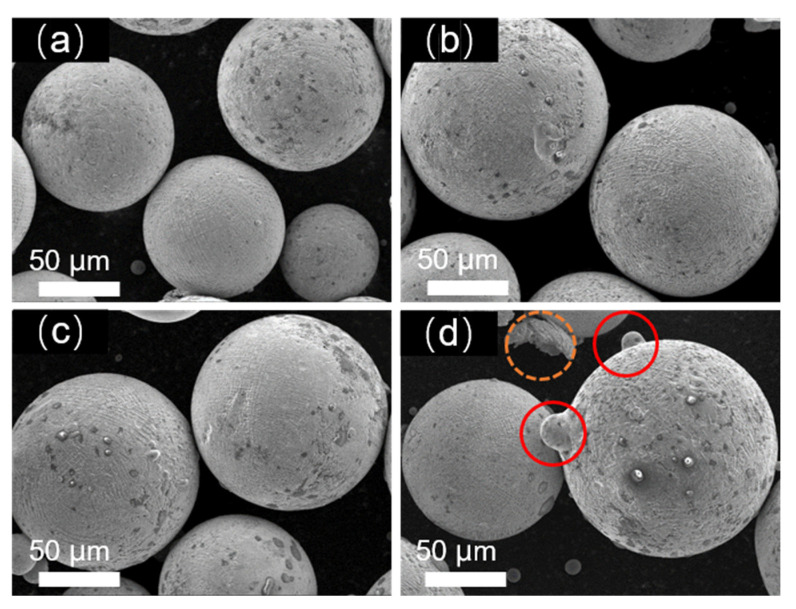
The morphological changes in Fe-Cr-Ni-W-B alloy powders under different atomization gas pressures. (**a**) 3.5 MPa, (**b**) 4 MPa, (**c**) 4.5 MPa, and (**d**) 5 MPa. Inside the orange circle is shaped powder, inside the red circle is satellite powder.

The atomization pressure directly determines the extent of the fragmentation and fracture behavior of the alloy melt, serving as the primary factor influencing the particle size of alloy powder. Therefore, this study first investigates and optimizes the atomization gas pressure in the gas atomization powder production process. Four different atomization pressures, namely 3.5 MPa, 4.0 MPa, 4.5 MPa, and 5.0 MPa, were selected for the research. Figure 1 depicts the morphology of Fe-Cr-Ni-W-B alloy powders prepared under different atomization pressures. It can be observed that all the as-prepared Fe-Cr-Ni-W-B alloy powders exhibited a predominantly spherical characteristic. However, with the increase in atomization pressure from 3.5 to 5.0 MPa, the smoothness of the powder surface gradually decreases. Additionally, the surface of large-particle powder begins to exhibit the adhesion of satellite particles with the increasing atomization pressure, as indicated by the red circles in the figures. At an atomization pressure of 5.0 MPa, as highlighted by the yellow circles in Figure 1d, the surface of the large-particle Fe-Cr-Ni-W-B alloy powder shows more adhesion of satellite particles, and some irregular particles were observed. Therefore, higher atomization pressure increases the irregularity of the powder surface.

**Figure 2:**
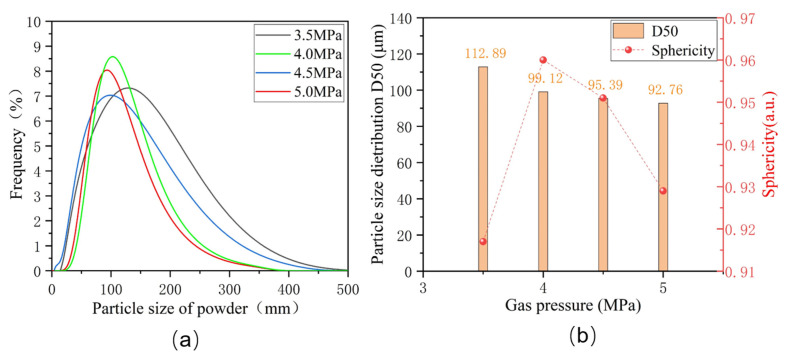
(**a**) The particle size distribution of alloy powders under different atomization pressures. (**b**) The particle size distribution and the degree of sphericity of the Fe-Cr-Ni-W-B powder under different atomization pressures.

Figure 2a displays the particle size distribution of Fe-Cr-Ni-W-B alloy powders under different atomization pressures. The particle sizes of alloy powders prepared under various pressures exhibit a single-peak distribution, indicating a thorough secondary fragmentation of the alloy melts during the atomization process. Figure 2b presents the D50 particle size distribution and the degree of the sphericity of Fe-Cr-Ni-W-B alloy powders under different atomization pressures. When the atomization pressure is 3.5 MPa, the D50 of the alloy powder is 112.89 μm. With the increase in atomization pressure to 4.0 MPa, the D50 of the atomized powder decreases to 99.12 μm. However, when the atomization pressure reaches 5 MPa, the D50 of the powder only decreases to 92.76 μm, and the further refinement of the powder becomes less significant. This is because when the atomization pressure is too high, the turbulence of the airflow and droplet particles inside the atomization chamber increases, and the small droplets of the atomized alloy melt gain greater kinetic energy. Before the small droplets completely solidify into spheres, they collide and merge to form satellite powders, causing the size of the alloy powder to coarsen. Overall, the average particle size of the alloy powder tends to decrease with an increase in atomization pressure.

According to the particle size equation proposed by Lubanska [20], the average particle sizes of the atomized alloy powders can be expressed as follows:

$$d_{m}{=d}_{L}K{\left[\frac{v_{m}}{v_{g}W_{e}}\left(1+\frac{M}{A}\right)\right]}^{0.5}$$

In the equation, *d*_m_ represents the average particle size of the atomized powder; *d*_L_ is the diameter of the metal melt (i.e., the nozzle diameter); K is a constant (typically between 40 and 50); *V*_m_ is the velocity of the metal melt; *V*_g_ is the velocity of the atomization gas; *M/A* is the mass flow ratio between the metal melt and the atomization gas; and *W*_e_ is the Weber number, which measures the atomization gas’s ability to break the melt, and based on this, determines the type of melt fragmentation. As the atomization pressure increases, the velocity *V*_g_ and kinetic energy of the atomization gas also significantly increase. The interaction between the airflow and the metal melt becomes more thorough, leading to a significant improvement in the efficiency of energy conversion between the atomization gas and the metal melt. Therefore, the alloy liquid is more easily broken into fine metal droplets over a given period, resulting in a reduction in the particle size of the alloy powder. Additionally, with the increase in the velocity *V*_g_ of the atomization gas, the Weber number *W*_e_ also increases, causing the fragmentation of the metal melt to transition from bag fragmentation to shear fragmentation [21]. As a result, the atomization and fragmentation effect on the alloy melt becomes more pronounced, leading to an increase in the fine powder content in the atomized alloy powder.

The sphericity test results for Fe-Cr-Ni-W-B alloy powders prepared under different atomization pressures are also presented in Figure 2b. At the lowest atomization pressure of 3.5 MPa, the Fe-Cr-Ni-W-B alloy powder delivers the lowest sphericity of 0.916. This is due to the relatively smaller surface area of these large particles, the uneven heat dissipation, and the more pronounced solidification shrinkage differences, which, during cooling, become more apparent, resulting in relatively lower sphericity of the powder. At atomization pressures of 4.0 MPa and 4.5 MPa, it is possible to achieve superior sphericity for alloy powders. As the atomization pressure increases up to 5 MPa, numerous smaller secondary dispersed particles adhere to the surface of the yet-to-solidify large droplets, thus forming satellite particles. This leads to a significant decrease in powder sphericity.

The flowability test results of alloy powders prepared under different atomization pressures are shown in Table 3. When the atomization pressure increases from 3.5 MPa to 4.5 MPa, the flowability of the powder improves, mainly due to a significant increase in powder sphericity. However, when the atomization pressure increases from 4.5 MPa to 5.0 MPa, the flowability of the powder slightly decreases. This is primarily because the number of satellite powders on the powder surface increases, or the small-diameter droplets aggregate and adhere to each other, forming agglomerates, leading to a decrease in powder sphericity. Meanwhile, the powder particle size further decreases, leading to increased friction and interlocking between the powder particles [22] and thus further reducing powder flowability. Therefore, the optimal atomization pressure range for Fe-Cr-Ni-W-B alloy powder atomization should be between 4.0 and 4.5 MPa. Considering the influence of atomization pressure on the particle size, sphericity, and flowability of alloy powders, as well as the consideration of improving atomization efficiency and reducing energy consumption, the optimal atomization pressure selected for this experiment is 4.5 MPa.

In conclusion, when the atomization pressure is small, the alloy melt will break into fountains [23,24]. After a certain distance from the diversion tube, the alloy melt is inhibited by the upward flow component, diffusing along the radial direction, and spreading towards the end of the diversion tube. The morphology is similar to that of a fountain. The outer alloy melt breaks up due to the dual influence of gas dynamics and its own gravity, and the molten metal droplets tear into strips. Under the action of surface tension, the banded metal melt slowly forms larger droplets. Because the atomization pressure is small, the gas impact force makes the alloy droplets obtain less surface energy, and the sphericity of the alloy powder is poor. When the atomization pressure is large, the liquid film of the alloy melt will break. After the alloy melt is discharged from the nozzle, it enters the return zone, and the upward flow along the atomization center produces a pressure gradient along the top of the nozzle, therefore forming a liquid film under the nozzle. The liquid film then forms a surface wave under the action of the surface tension and the kinematical viscosity of the atomized gas, and the surface wave then develops into a short wave until the unstable rupture forms tiny melt drops. Due to the large atomization pressure, the gas impact force causes the alloy droplets to obtain a large surface energy, and the sphericity of the alloy powder is improved. When the atomization pressure is too large, the probability of collision between small droplets increases under the action of a strong impact force. Therefore, more satellite balls will be formed, thus reducing the sphericity of the powder.

### 3.2. The Effect of the Inner Diameter of the Melt Nozzle on the Flowability of the Fe-Cr-Ni-W-B Powder

**Figure 3:**
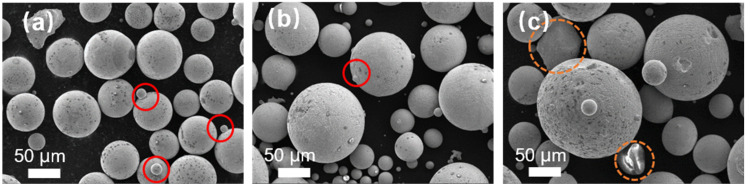
The morphology of alloy powders prepared under different inner diameters of the melt nozzles. (**a**) 5.0 mm; (**b**) 5.3 mm; (**c**) 5.6 mm. Inside the orange circle is shaped powder, inside the red circle is satellite powder.

To investigate the influence of the inner diameter of the melt nozzle on alloy powders during atomization, the diameter of the melt nozzle was adjusted to alter the flow rate of the molten metal, while maintaining the atomization pressure at 4.5 MPa. Figure 3 illustrates the SEM image of Fe-Cr-Ni-W-B alloy powders prepared under different atomization inner diameters of the melt nozzles. When the inner diameter of the melt nozzles is 5.0 mm, there are more satellite particles on the surface of the alloy powder, as indicated by the red circle in Figure 3a. With a smaller inner diameter of the melt nozzle, the flow rate of the alloy melt through the melt nozzles is reduced over time. Under the same atomization pressure, the reduced inner melt nozzle diameter leads to the dispersion of the alloy melt into more secondary dispersed droplets. The increased probability of collisions between these droplets results in the formation of a larger number of satellite particles.

With an increase in the inner diameter of the melt nozzle up to 5.3 mm, the number of satellite powders in the powder decreases, as indicated by the red circles in Figure 3b. This is because, with a smaller tundish flow control tube aperture, there are more secondary dispersed droplets in the atomization zone, leading to an increased collision probability between droplets and the formation of a certain amount of satellite powders. However, when the inner diameter of the melt nozzle increases to 5.6 mm, irregularly shaped powders appear, as highlighted by the yellow circles in the Figure 3c. With a tundish flow melt nozzle aperture of 5.6 mm, the breakup efficiency of the melt per unit volume is lower, and the secondary dispersion of some sheet-like or ribbon-like droplets is insufficient, resulting in direct cooling and, in turn, the solidification into sheet-like powders, thereby affecting the powder sphericity. This is because when the aperture of the melt nozzle is larger, there are more droplets in the atomization area, which leads to a lower efficiency of melt fragmentation per unit volume. As a result, the secondary fragmentation of some flake- or ribbon-like droplets is insufficient, and they directly cool and solidify, forming a flake-like powder.

**Figure 4:**
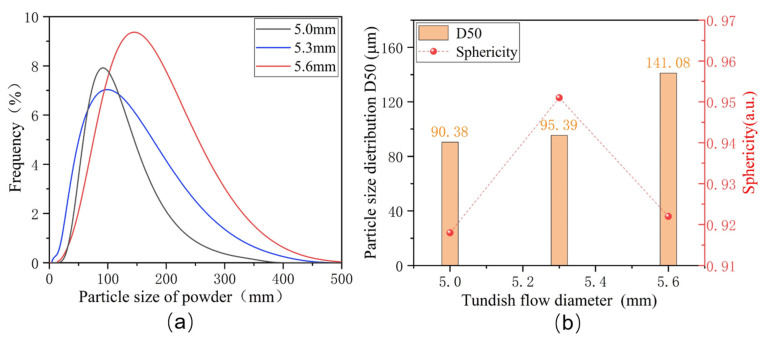
(**a**) Particle size distribution of alloy powders under the different inner diameters of the melt nozzles. (**b**) The D50 particle size distribution and the degree of sphericity of Fe-Cr-Ni-W-B powders.

Figure 4a,b illustrate the particle size distribution of alloy powders prepared under the different atomization inner diameters of the melt nozzles. As the inner diameter of the melt nozzles increases from 5.0 mm to 5.6 mm, the average particle size of the Fe-Cr-Ni-W-B alloy powder gradually increases. When the inner diameter of the melt nozzle is 5.0 mm, the D50 of the alloy powder is 90.39 μm. With the increase in aperture to 5.3 mm, the D50 increases to 95.39 μm. When the tundish flow control tube aperture is 5.6 mm, the D50 of the alloy powder increases to 141.08 μm. As the diameter of the melt nozzle decreases, the resistance encountered by the molten metal increases, resulting in a slower flow velocity. Consequently, within a unit volume, the collision efficiency between the atomized gas and the alloy melt increases, leading to better fragmentation effects and the attainment of finer powder particle sizes.

Figure 4b also displays the sphericity of alloy powders prepared under different diameters of the melt nozzles. It can be observed that, with the diameter of the melt nozzle at 5.3 mm, the sphericity of the powder reaches the highest value. Therefore, by comprehensively considering various factors, this study determines the optimal tundish flow control tube diameter to be 5.3 mm. With further reductions in the diameter of the melt nozzle to 5.0 mm, the number of the droplets of the alloy melt in the atomization region decreases significantly. This leads to an increased probability of collisions between droplets, resulting in the formation of numerous secondary fine particles. These secondary particles adhere to the surface of the alloy powder, causing a significant decrease in sphericity.

Table 4 highlights that, when the melt nozzle inner diameter increases from 5.0 mm to 5.3 mm, the flowability of the alloy powder improves, changing from 16.39 (s/50 g) to 15.88 (s/50 g). This is mainly due to the effective enhancement of the powder sphericity. However, when the melt nozzle inner diameter increases to 5.6 mm, the flowability decreases. This is because the number of flake- and irregular-shaped powders within the powder increases.

In summary, when the melt nozzle inner diameter is small, the falling resistance of the metal melt increases, the flow speed slows down, the collision efficiency of the atomized gas and alloy melt per unit volume is higher, the crushing effect is better, and the particle size of the obtained powder is smaller. When the melt nozzle inner diameter is too small, the probability of collisions between small droplets per unit volume increases greatly, resulting in the formation of more satellite particles, worsening the sphericity of the powder particles. When the melt nozzle inner diameter is large, the falling resistance of the metal melt decreases, the flow speed increases, the melt crushing efficiency per unit volume is low, the crushing effect is weakened, the kinetic energy obtained by the melt drops decreases, the sphericity of the powder is not complete, the secondary crushing effect of some flaking or ligamentary droplets is insufficient, and the flaking powder is formed via direct cooling and solidification. At this time, the particle size of the powder is large, and the sphericity of the powder is poor. Therefore, the alloy powder with a small average particle size and good sphericity can be obtained with the appropriate melt nozzle inner diameter. Therefore, the optimal melt nozzle inner diameter for the atomization of the Fe-Cr-Ni-W-B alloy powder should be 5.3 mm.

**Figure 5:**
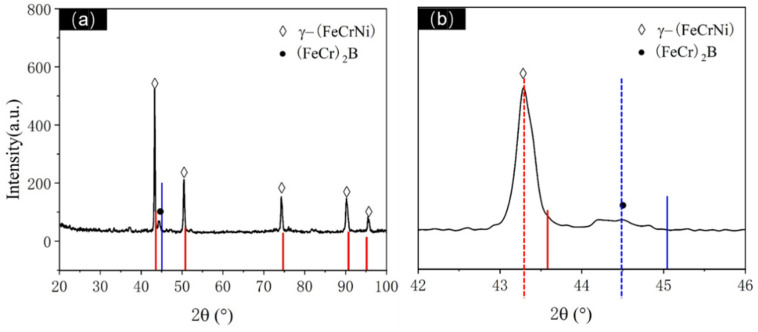
(**a**) XRD diffraction pattern of atomized Fe-Cr-Ni-W-B powder. (**b**) Enlarged XRD patterns at 42~46°. The solid red line represents the standard diffraction peak of γ-(FeCrNi), the solid blue line represents one of the standard diffraction peak of (FeCr)_2_B. The dashed lines represent the actual diffraction peak of the corresponding phase.

XRD analysis was conducted on the atomization-prepared Fe-Cr-Ni-W-B alloy powder with an atomization pressure of 4.5 MPa and a nozzle diameter of 5.3 mm, as shown in Figure 5. Post-atomization, the matrix phase in the alloy powder is identified as being γ-(FeCrNi) phase containing W (PDF 00-035-1375). The diffraction peaks of this phase exhibit a low-angle shift and an increase in the lattice constant. This is attributed to the rapid solidification cooling rate during atomization, where the alloy melt is dispersed into fine droplets via gas. The diffusion rate of W atoms, due to their larger atomic radius, is relatively slow, thus preventing complete diffusion. As a result, most W atoms are oversaturated and solid-solved within the matrix, leading to an increase in the lattice constant. Additionally, a small amount of boride phase (FeCr)_2_B (PDF 97-001-6809) is also present in the sample. From Figure 5b, it can be observed that the strongest peak of the (FeCr)_2_B phase appears at around 45°, and the diffraction peaks of the (FeCr)_2_B phase are significantly shifted towards lower angles, indicating that most W atoms are solid-solved within the borides, resulting in an increase in the lattice constant for this phase as well.

**Figure 6:**
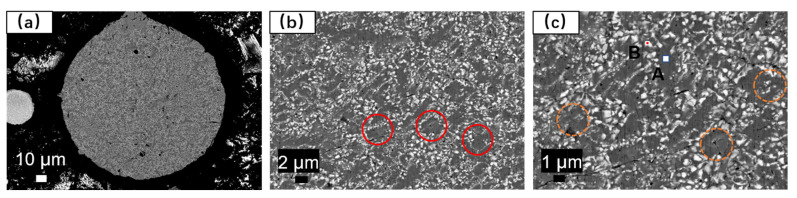
Cross-sectional BESEM and EDS analysis of gas-atomized Fe-Cr-Ni-W-B spherical alloy powders, where (**a**–**c**) represent cross-sectional BESEM images. In the red circle is a discontinuous network structure. The orange circle indicates that the borides are located in the crystal.

The cross-sectional analysis of the alloy powder was conducted using BESEM and EDS, as depicted in Figure 6. The elemental composition of localized microregions in the alloy powder is presented in Table 5. The γ-(FeCrNi) matrix phase containing W exhibits a size ranging from 2–5 µm. The white regions represent (FeCr)_2_B borides containing W, dispersed in a point- or chain-like manner around the gray matrix, without exhibiting a continuous mesh distribution, as indicated by the red circle in Figure 6b. Additionally, boride sizes at submicron scales are unevenly distributed, with some borides being located within the matrix grains, as highlighted by the yellow circles in Figure 6c.

**Figure 7:**
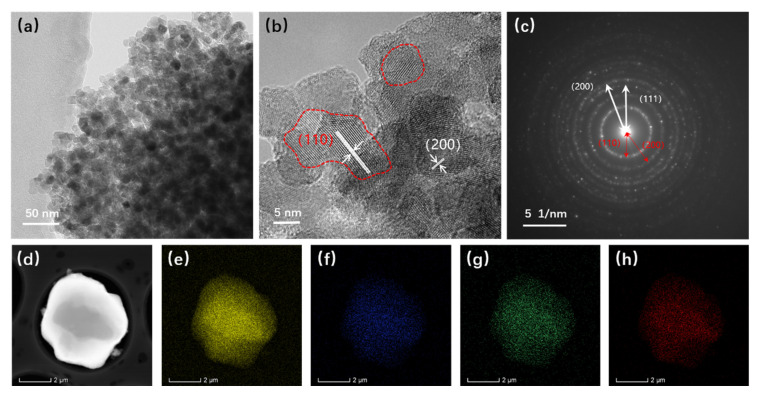
TEM images of atomized Fe-Cr-Ni-W-B powders. (**a**) High-magnification TEM images. (**b**) HRTEM images. (**c**) SAED images. (**d**) Low-magnification TEM images. (**e**–**h**) EDS mapping of W, Ni, Fe, and Cr elements, respectively.

The structure of the atomized Fe-Cr-Ni-W-B spherical powder was further examined using TEM, and the results are illustrated in Figure 7. In Figure 7a, it is evident that the atomized Fe-Cr-Ni-W-B spherical alloy powder comprises numerous nanocrystalline particles with sizes ranging from 5 to 10 nm. This is primarily attributed to the rapid cooling of the alloy droplets during the aerosolization process, which induces substantial supercooling and a significant increase in the nucleation rate. The HRTEM image (Figure 7b) reveals that the (110) crystal plane of the Fe_2_B phase has a crystal plane spacing of *d* = 3.61, and the (200) crystal surface has a crystal plane spacing of *d* = 1.81. Based on the results of an SAED study of the area outside the red box (Figure 7c), we can deduce that this is composed of polycrystalline diffraction rings, two of which correspond to the (110) and (200) crystal planes of the Fe_2_B phase, and the third corresponds to the (111) crystal plane of the Fe_0.7_Cr_0.19_Ni_0.11_ phase, thus matching well with the XRD results. The EDS mapping (Figure 7e–h) further shows that the atomized Fe-Cr-Ni-W-B spherical powder produced using the aerosolization approach maintains a consistent composition throughout, with no discernible segregation between the various components. Therefore, the gas atomization method can effectively eliminate the reticulation structure from the raw powder material, enabling the preparation of high-performance Fe-Cr-Ni-W-B bulk materials with a high degree of sphericity.

### 3.3. The Effect of the Heat Treatment Temperature on the Flowability of the Fe-Cr-Ni-W-B Powder

**Figure 8:**
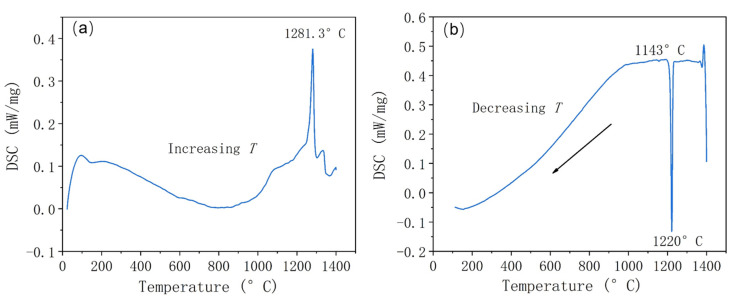
The DSC curves of the atomized Fe-Cr-Ni-W-B spherical powder. (**a**) DSC heating curve at 10 °C/min. (**b**) DSC cooling curve at 10 °C/min.

The Fe-Cr-Ni-W-B powder underwent heat treatment following atomization. Figure 8 shows the DSC heating and cooling curves of the atomized Fe-Cr-Ni-W-B alloy powder. In Figure 8a, the heating curve exhibits an endothermic peak at 1281 °C, corresponding to the eutectic reaction temperature of borides within the alloy powder. In the cooling curve, a prominent endothermic peak appears at 1143 °C, corresponding to the precipitation of borides. Therefore, the heat treatment temperature for the Fe-Cr-Ni-W-B alloy powder is set to around 1150 °C.

**Figure 9:**
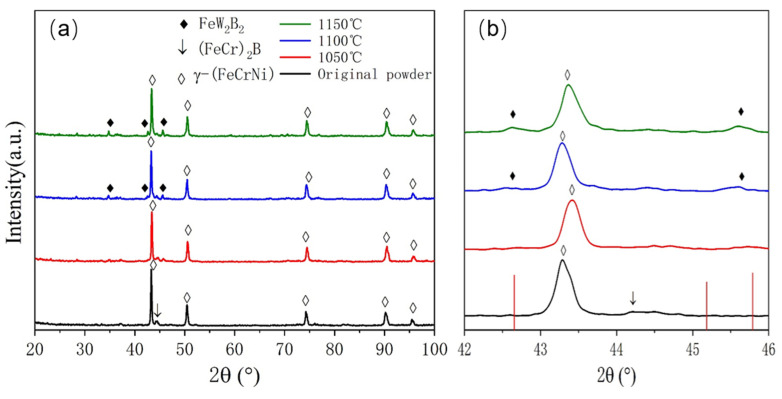
(**a**) XRD patterns of atomized Fe-Cr-Ni-W-B spherical powder after vacuum heat treatment for 1 h at different temperatures. (**b**) Enlarged XRD patterns at 42–46°.

The phase evolution of the Fe-Cr-Ni-W-B alloy powder at different temperatures following vacuum heat treatment for 1 h is shown in Figure 9. When compared to the original powder, following the heat treatment at 1050 °C, both the strength of the matrix phase γ-(FeCrNi) and the second phase (FeCr)_2_B in the alloy powder decreased. When the heat treatment temperature is raised to 1100 °C, the W atoms in the γ-(FeCrNi) matrix obtain enough energy and have sufficient time to diffuse into (FeCr)_2_B, replacing the Fe/Cr atoms. When the W atoms in (FeCr)_2_B reach a certain amount, the phase transition occurs and the FeW_2_B_2_ (PDF#00-021-0427) phase appears. Since Fe and Cr are miscible, and a certain amount of Cr is detected in the FeW_2_B_2_ phase, it is believed that the actual boron-containing phase is the (FeCr)W_2_B_2_ phase. As the heat treatment temperature increases to 1150 °C, the intensity of the diffraction peaks of the (FeCr)W_2_B_2_ phase in the powder continuously increases also, indicating an increasing content of this phase.

**Figure 10:**
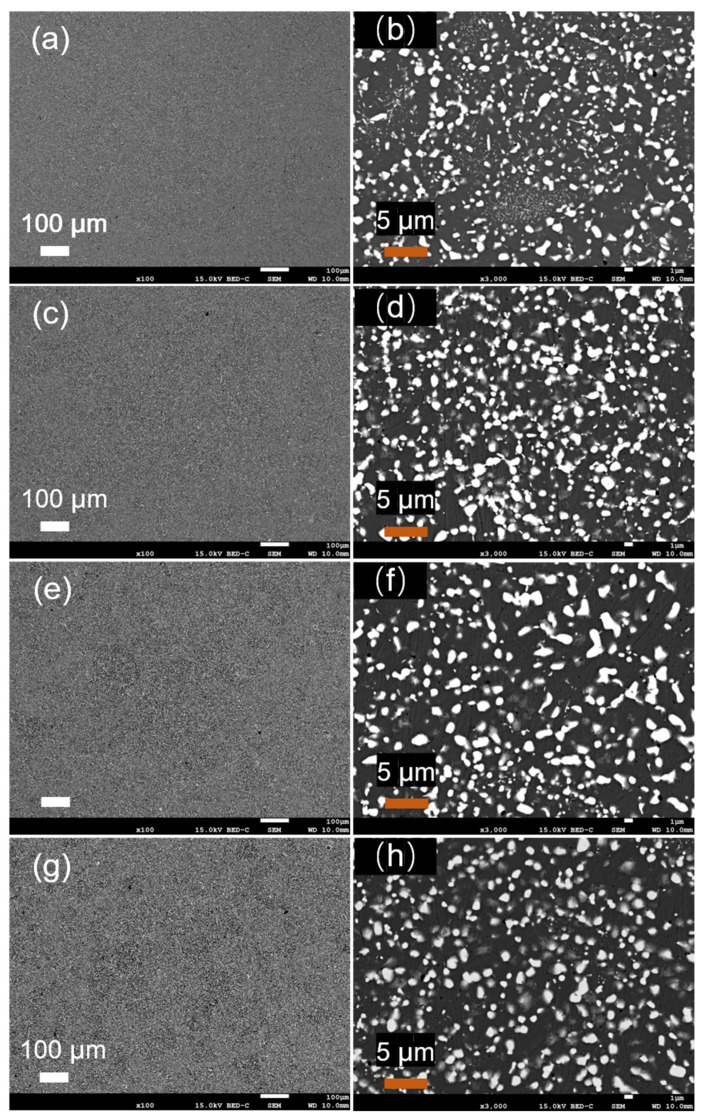
Microscopic morphology of the atomized Fe-Cr-Ni-W-B spherical powder following the vacuum heat treatment for 1 h at different temperatures, where (**a**) and (**b**) are untreated; (**c**) and (**d**) are at 1050 °C; (**e**) and (**f**) are at 1100 °C; and (**g**) and (**h**) are at 1150 °C.

To analyze in detail the effect of the heat treatment temperature on the microstructure of the atomized Fe-Cr-Ni-W-B alloy powder, the microstructure of the powder following the heat treatment for 1 h at different temperatures was characterized, as shown in Figure 10. When compared to the alloy powder after atomization (Figure 10a,b), the white borides in the alloy powder gradually transformed from a granular distribution to a reticular distribution. When the heat treatment temperature reached 1100 °C, the reticular structure of the white borides in the powder became more pronounced, and the local aggregation of white borides even occurred, forming aggregates on a micrometer scale. As the temperature continued to rise to 1150 °C, both the number and scale of boride aggregates increased.

**Figure 11:**
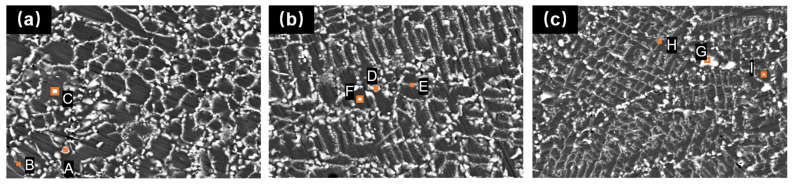
The cross-sectional EDS results of the Fe-Cr-Ni-W-B powder after 1 h of the vacuum heat treatment at different temperatures. (**a**) 1050 °C, (**b**) 1100 °C, (**c**) 1150 °C.

Figure 11 shows the EDS analysis results of the Fe-Cr-Ni-W-B powder after the vacuum heat treatment for 1 h at different temperatures, and Table 6 displays the corresponding atomic percentage contents in the marked analysis areas. The results indicate that the bright areas are rich in W and B elements, which, combined with the XRD results in Figure 9, corresponds to the (FeCr)W_2_B_2_ phase. Additionally, numerous small gray-white particles appear around the (FeCr)W_2_B_2_ phase. The EDS characterization of areas in Figure 11(B, E, H) shows that these regions have a high content of W atoms, but they do not reach the stoichiometric ratio of (FeCr)W_2_B_2_, indicating that these areas are transition regions. As the heat treatment temperature increases, W atoms from the matrix fully diffuse into these transition regions, leading to their transformation into the (FeCr)W_2_B_2_ phase. Therefore, during the heat treatment, a large amount of W atoms dissolved in the matrix enter the (FeCr)_2_B phase, causing it to transform into the (FeCr)W_2_B_2_ phase. Compared to the (FeCr)_2_B phase, the (FeCr)W_2_B_2_ phase has a significantly increased density and a sharp volume contraction, resulting in the appearance of broken networks of borides distributed along the grain boundaries of the matrix, thus disrupting their continuous distribution and weakening the brittle borides’ segmentation of the matrix. This would improve the ductility of the alloy material following powder metallurgy.

**Figure 12:**
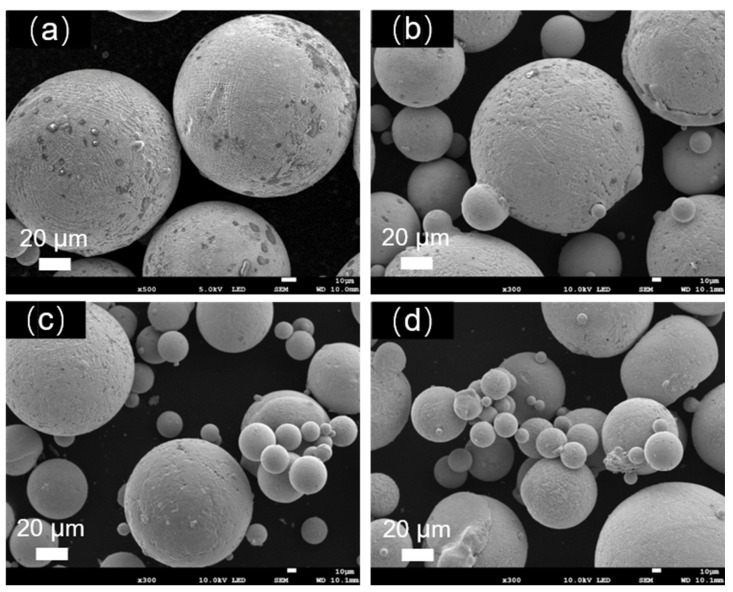
SEM micrographs of the gas-atomized Fe-Cr-Ni-W-B spherical powder after the vacuum heat treatment for 1 h at different temperatures, where (**a**) is untreated; (**b**) is at 1050 °C; (**c**) is at 1100 °C; (**d**) is at 1150 °C.

Figure 12 illustrates the SEM morphology of the alloy powder before and after the heat treatment. It can be observed that, after the heat treatment, many smaller powder particles agglomerate together or adhere to the surface of larger particles, forming satellite powders. With the increase in the heat treatment temperature, the phenomenon of powder agglomeration on the surface becomes more pronounced. Table 7 presents the changes in the particle size distribution, flowability, and bulk density of the Fe-Cr-Ni-W-B powder following the vacuum heat treatment for 1 h at different temperatures. It can be seen that the D50 value of the alloy powder increases after the heat treatment, while both the flowability and bulk density decrease to some extent. This is related to the changes in the surface morphology and sphericity of the alloy powder after the heat treatment. Due to the larger surface area of smaller powder particles, their melting point decreases, leading to a phenomenon where they re-melt during the heat treatment, resulting in a mutual connection between powder particles and the formation of sintering necks. This phenomenon significantly reduces the sphericity, flowability, and bulk density of the powder.

In summary, while obtaining alloy powders containing the (FeCr)W_2_B_2_ phase, it is essential to ensure the flowability of the powder as much as possible; this will ensure that the alloy powder can adequately fill the mold cavity during powder metallurgy. Therefore, it is recommended that the heat treatment temperature is set to 1100 °C. If further improvement in the flowability of the alloy powder after the heat treatment is desired, adequate fragmentation methods can be employed.

**Figure 13:**
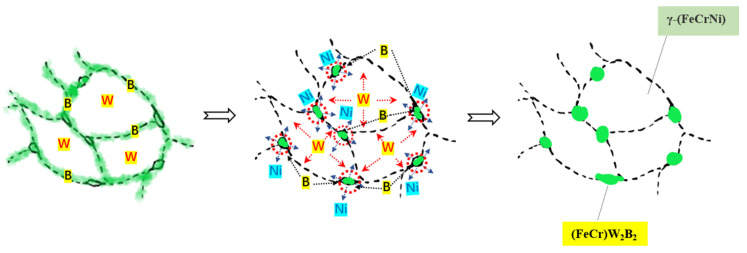
The process of atomic diffusion and phase evolution.

Due to the low diffusion activation energy Q_B_ of the B atom in γ-Fe, its diffusion coefficient D_b_ is high. Therefore, in the process of aerosol solidification, the B atoms rapidly diffuse to the austenite grain boundary, and the (FeCr)_2_B phase is formed. However, the activation energy of the W atom in γ-Fe is high, and its diffusion rate is slow. In the state of rapid solidification, only a small part of the W atoms are sonically dissolved in the (FeCr)_2_B phase, and most of the W atoms are still retained in γ-(FeCrNi) to form saturated solid solutions. The higher energy of the grain boundary provides energy for the nucleation of the second phase. Following the heat treatment, the large size (FeCr)W_2_B_2_ particles of the powder are first formed in the junction area of several austenite grains. Figure 13 shows the process of atomic diffusion and phase evolution. The W atoms diffuse from the inside of austenite towards the interface, and the W atoms at the interface further diffuse towards the interface region of multiple austenite grains, resulting in the highest W content being in the interface region of multiple austenite grains, where the (FeCr)W_2_B_2_ phase is formed earliest, and the second phase is formed according to the spherical growth with the lowest interface energy. Therefore, the formed (FeCr)W_2_B_2_ phase is distributed in a granular manner, and these particles will grow with the increase in the heat treatment temperature or the extension of the heat treatment time. At the same time, with the precipitation of (FeCr)W_2_B_2_, the content of W in the second phase continues to increase, the density continues to increase, the volume shrinks, the boride network structure breaks off, and local chain distribution is formed. In addition, the Ni atoms in the borides reverse diffuse into the matrix, thus strengthening and stabilizing the austenite phase. Therefore, the microstructure of the Fe-Cr-Ni-W-B spherical powder can be regulated by the heat treatment, so that the second phase (FeCr)W_2_B_2_ in the powder is precipitated in a finely dispersed particle size. It can effectively solve the difficult problem of boride network distribution. The laws are of utmost significance, as the chain distribution of the (FeCr)W_2_B_2_ phase in powder metallurgy or additive manufacturing post-formation facilitates the attainment of high-quality components with enhanced strength and toughness.

## 4. Conclusions

This study employed the gas atomization process to fabricate a high-quality Fe-Cr-Ni-W-B alloy powder. It systematically investigated the effects of atomization gas pressure, nozzle diameter, and heat treatment temperature on the flowability of the Fe-Cr-Ni-W-B powder, aiming to prepare an alloy powder with excellent flowability, laying the groundwork for the fabrication of a high-strength and tough Fe-Cr-Ni-W-B alloy. Through the optimization of process parameters, an alloy powder with suitable properties was obtained, using an atomization pressure of 4.5 MPa and a nozzle diameter of 5.3 mm resulted in a spherical powder with a sphericity of 95.1%, a flowability of 15.88 s/50 g, and a D50 of 95.39 µm. Subsequently, after the heat treatment at 1100 °C for 1 h, the alloy powder exhibited a D50 of 149.0 µm, and a flowability of 17.83 s/50 g, featuring a γ-(FeCrNi) matrix and (FeCr)W_2_B_2_ as the second phase, thereby establishing a foundation for the preparation of a high-strength and tough Fe-Cr-Ni-W-B alloy.

## Figures and Tables

**Table 1:** Main chemical composition of the raw material.

Raw Powders	Standard	W (wt.%)	Cr (wt.%)	B (wt.%)	Ni (wt.%)	Fe (wt.%)	M (wt.%)
Fe-W alloy	FeW_80_	80.91	/	/	/	18.38	0.71
Fe-Cr alloy	FeCr_55_	/	59.50	/	/	39.40	1.10
Fe-B alloy	FeB_20_	/	/	19.84	/	79.35	0.81
Pure Ni	Ni200	/	/	/	99.90	/	0.10
Pure iron	DT4	/	/	/	/	99.78	0.22

**Table 2:** Process parameters of the gas atomization.

Parameters	Values
Atomization pressure (MPa)	3.5~5
Atomization temperature (°C)	1700
The inner diameter of the melt nozzle (mm)	5.0~5.6
Atomization medium	Argon

**Table 3:** Flowability of Fe-Cr-Ni-W-B alloy powders at different atomization gas pressures.

Atomization Gas Pressure (MPa)	Flowability (s/50 g)
3.5	16.14 ± 0.09
4.0	15.76 ± 0.05
4.5	15.88 ± 0.06
5.0	16.52 ± 0.11

**Table 4:** Flowability test results of alloy powders prepared under different melt nozzle inner diameters.

Melt Nozzle Inner Diameter (mm)	Flowability (s/50 g)
5.0	16.39 ± 0.11
5.3	15.88 ± 0.06
5.6	16.02 ± 0.08

**Table 5:** Elemental composition of microregions in gas-atomized Fe-Cr-Ni-W-B spherical powders.

Zones	Element					
	Cr (at. %)	Fe (at. %)	Ni (at. %)	W (at. %)	B (at. %)	Total
A	14.15	69.39	12.14	4.04	/	100
B	18.91	31.37	3.76	13.87	32.10	100

**Table 6:** The atomic percentage content of micro-regions in the Fe-Cr-Ni-W-B powder after 1 h of the vacuum heat treatment at different temperatures.

Temperature	Zones	Elements				
		B (at. %)	W (at. %)	Fe (at. %)	Cr (at. %)	Ni (at. %)
1050 °C	A	34.03	16.97	26.02	20.83	2.14
	B	21.05	8.71	47.10	16.02	7.12
	C	-	3.81	69.95	13.64	12.61
1100 °C	D	43.35	23.61	16.27	15.24	1.52
	E	31.36	9.55	37.78	16.17	5.14
	F	-	3.64	69.89	14.43	12.04
1150 °C	G	41.48	28.85	15.61	16.11	0.94
	H	18.59	7.72	50.41	15.63	7.72
	I	-	2.94	70.59	14.15	12.32

**Table 7:** Changes in particle size distribution, flowability, and density of the alloy powder before and after the heat treatment.

Powder State	D_50_ (µm)	Flowability (s/50 g)	Tap Density (g/cm^3^)
Before treatment	95.39 ± 0.04	15.88 ± 0.06	5.11 ± 0.03
1050 °C	140.0 ± 0.02	16.56 ± 0.12	5.00 ± 0.03
1100 °C	149.0 ± 0.04	17.83 ± 0.14	4.85 ± 0.04
1150 °C	160.40 ± 0.06	18.77 ± 0.15	4.80 ± 0.03

## Data Availability

Data are contained within the article.
